# Exploring the Impact of Cerebrovascular Disease and Major Depression on Non-diseased Human Tissue Transcriptomes

**DOI:** 10.3389/fgene.2021.696836

**Published:** 2021-07-19

**Authors:** Chi-Lam Poon, Cho-Yi Chen

**Affiliations:** ^1^Institute of Biomedical Informatics, National Yang Ming Chiao Tung University, Taipei, Taiwan; ^2^Weill Cornell Graduate School of Medical Sciences, Cornell University, New York, NY, United States; ^3^Brain Research Center, National Yang Ming Chiao Tung University, Taipei, Taiwan

**Keywords:** complex disease, cerebrovascular disease, major depression, inflammation, transcriptome, GTEx

## Abstract

**Background:**

The development of complex diseases is contributed by the combination of multiple factors and complicated interactions between them. Inflammation has recently been associated with many complex diseases and may cause long-term damage to the human body. In this study, we examined whether two types of complex disease, cerebrovascular disease (CVD) or major depression (MD), systematically altered the transcriptomes of non-diseased human tissues and whether inflammation is linked to identifiable molecular signatures, using post-mortem samples from the Genotype-Tissue Expression (GTEx) project.

**Results:**

Following a series of differential expression analyses, dozens to hundreds of differentially expressed genes (DEGs) were identified in multiple tissues between subjects with and without a history of CVD or MD. DEGs from these disease-associated tissues—the visceral adipose, tibial artery, caudate, and spinal cord for CVD; and the hypothalamus, putamen, and spinal cord for MD—were further analyzed for functional enrichment. Many pathways associated with immunological events were enriched in the upregulated DEGs of the CVD-associated tissues, as were the neurological and metabolic pathways in DEGs of the MD-associated tissues. Eight gene-tissue pairs were found to overlap with those prioritized by our transcriptome-wide association studies, indicating a potential genetic effect on gene expression for circulating cytokine phenotypes.

**Conclusion:**

Cerebrovascular disease and major depression cause detectable changes in the gene expression of non-diseased tissues, suggesting that a possible long-term impact of diseases, lifestyles and environmental factors may together contribute to the appearance of “transcriptomic scars” on the human body. Furthermore, inflammation is probably one of the systemic and long-lasting effects of cerebrovascular events.

## Introduction

Complex diseases are caused by genetic, environmental and lifestyle factors and their interactions, most of which have not yet been identified. Recent studies have revealed that the immune system and inflammatory responses are involved in a wide range of complex diseases, such as cardiovascular disease (Willerson and Ridker, 2004), stroke (Anrather and Iadecola, 2016), cancer (Coussens and Werb, 2002), and psychiatric disorders (Yuan et al., 2019). Inflammation is generally defined as the immune system’s response that defends against injury or stress (Netea et al., 2017). In a normal inflammatory response, the upregulation of inflammatory activity is strictly regulated. However, with psychological, environmental and biological factors (Schneiderman et al., 2005; Sears and Genuis, 2012; Zhu et al., 2014; Furman et al., 2019), the regulated process can become uncontrolled in the resolution phase, causing a systemic chronic inflammation that contributes to damage in all tissues and organs and increases the risk of diseases that remain global leading causes of disability and mortality (Murray and Lopez, 1997; Virani et al., 2020).

The Genotype-Tissue Expression (GTEx) project (Aguet et al., 2020) has established a database of expression data, whole genome sequences, and whole-exome sequences of 54 non-diseased tissue sites across nearly 1,000 individuals (as of the current v8 release). Genotype-Tissue Expression also collected subject phenotype data, including demographic information, general medical histories, histories at the time of death, the circumstances of death, and so on. The medical histories were provided by the hospital systems, which recorded the prior care of deceased donors.

Here we evaluate whether past chronic inflammatory diseases could leave biological alterations (“scars”) in non-diseased tissues of the human. We have focused on two types of complex disease related to the brain, cerebrovascular disease (CVD) and major depression (MD), as they are typical chronic inflammatory diseases with heritable components (Flint and Kendler, 2014; Boehme et al., 2017; Dichgans et al., 2019; Ormel et al., 2019), and there are enough cases of these in the GTEx database. CVD comprises clinical conditions that impair blood flow to the brain, including strokes, transient ischemic attacks, intracranial aneurysms, and other vessel diseases (Goldstein and Lichtman, 2013). Major depression is one of the most common psychiatric illnesses, ranging from 3 to 16.9 percent worldwide (Kessler et al., 2003; Demyttenaere et al., 2004), and has a significant impact on society. It is characterized by a persistent feeling of sadness or a loss of interest or pleasure in outside stimuli. Previous large-scale genome-wide association studies (GWAS) and meta-analyses have identified a large number of genetic loci associated with stroke (Malik et al., 2018; Keene et al., 2020) and depression (Wray et al., 2018; Howard et al., 2019; Ormel et al., 2019) in multi-ancestry groups. However, genetic variability contributing to the susceptibility mechanism underlying CVD and depression as well as their interactions with inflammation remains not fully identified or characterized.

In this study, we aimed to answer the following two questions: (1) is there any significant transcriptomic difference in non-diseased tissues with and without a history of CVD or MD? (2) if yes, is there any evidence to indicate that inflammation may play a role in shaping these transcriptomic landscapes? We performed a differential expression analysis on each GTEx tissue by comparing the expression profiles between subjects with and without the medical history of CVD or MD. Top differentially expressed genes (DEGs) identified in multiple tissues from the series of DE analyses were included in the downstream functional enrichment analysis. We also performed transcriptome-wide association studies (TWAS) on inflammation biomarkers to find any overlaps with the DEGs.

## Results

### Cohorts and Risk Factors

Multi-tissue RNA-seq data were compiled from the GTEx project, as described in the Materials and Methods section. Subjects with an explicitly reported medical history of cerebrovascular disease or major depression were considered in this study. A total of 16,412 samples across 46 tissues, obtained from 928 subjects, were included in the CVD analysis (Supplementary Figure 1), and 16,221 samples across 45 tissues from 926 subjects in the MD analysis (Supplementary Figure 2).

Risk factors of complex diseases include clinical variables such as age (Niccoli and Partridge, 2012), sex (Ober et al., 2008), and BMI (Knight, 2011). The average age of the cohort with a history of CVD was significantly higher than that of the non-CVD cohort (CVD 52.09 ± 13.09, non-CVD 57.74 ± 10.79 yrs, *P* = 3.87 × 10^–6^, *t*-test), while BMI, sex and race showed no significant differences between the two groups (Supplementary Figure 3). The average age of the MD cohort was younger than that of the non-MD cohort (MD 49.69 ± 13.33, non-MD 53.07 ± 12.91 yrs, *P* = 1.82 × 10^–2^, *t*-test). Moreover, females had a higher incidence of developing depression (*P* = 0.01, Chi-squared test) (Supplementary Figure 4). This is consistent with the higher prevalence of major depressive disorder in women than in men (Kessler, 2003). To eliminate possible confounding effects from these risk factors, we included them as covariates into our multivariate linear models in the differential expression analysis.

### Differentially Expressed Genes Identified in Multiple Tissues

To investigate whether past CVD or MD left “transcriptomic scars” on any tissues or organs, we implemented the voom-limma (Law et al., 2014; Ritchie et al., 2015) pipeline to identify genes differentially expressed between the cohorts with and without a history of CVD or MD, using the linear model described in the Materials and Methods section.

**FIGURE 1:**
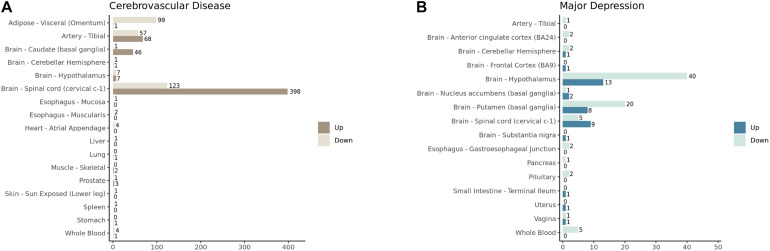
The number of significantly upregulated and downregulated genes (FDR < 0.05 and absolute log2 fold change > 1.5) identified in GTEx tissues in **(A)** cerebrovascular diseases and **(B)** major depression analyses.

Since the analyzing tissues were defined as non-diseased, we did not expect that there would be a significant number of DEGs identified. To our surprise, 17 out of 46 and 16 out of 45 tissues displayed significant differential expression (false discovery rate <0.05 and absolute fold change >1.5) in the CVD and MD analyses, respectively (Figures 1A,B). The top four tissues with the highest number of significant DEGs were included in the functional enrichment analysis (Adipose – Visceral, Artery – Tibial, Brain – Caudate, and Brain – Spinal cord for CVD), as well as the top three MD tissues (Brain – Hypothalamus, Brain – Putamen, and Brain – Spinal cord).

**FIGURE 2:**
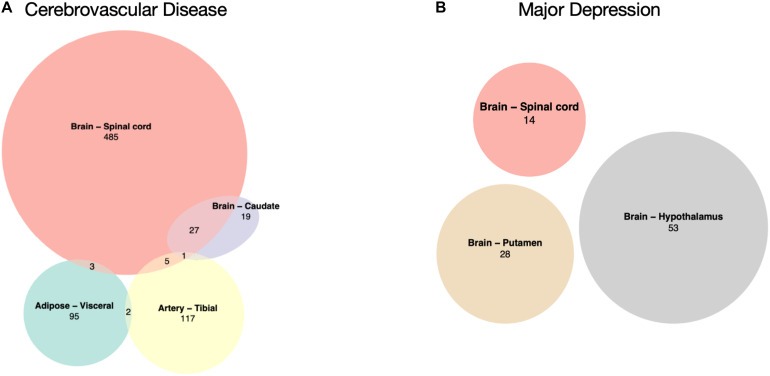
Overlaps of significantly differentially expressed genes. **(A)** CVD-associated tissues: Adipose – Visceral, Artery – Tibial, Brain – Caudate, and Brain – Spinal cord. **(B)** MD-associated tissues: Brain – Hypothalamus, Brain – Putamen, and Brain – Spinal cord, there is no overlap between these three.

There was no common DEG shared by the four CVD or the three MD tissues (Figures 2A,B). A large number of significant DEGs identified by our CVD model (Supplementary Table 1) were associated with inflammation. For instance, the most significantly upregulated gene in the spinal cord, *CHI3L1*, is related to a variety of inflammatory disorders (Kastrup et al., 2009; Johansen et al., 2010; Im et al., 2020) and coronary artery disease (Rathcke and Vestergaard, 2009). Gene *FCGR3A*—upregulated in three of the CVD tissues (Brain – Spinal cord adj.P.val = 0.02; Brain – Caudate adj.P.val = 0.03; Artery – Tibial adj.P.val = 0.02)—encodes a receptor that binds the Fc portion of IgG, and it affects the pharmacokinetics in patients with Crohn’s Disease (Termant et al., 2015). *LPAR5*, which was overexpressed in both the brain caudate and the spinal cord of the subjects with a history of CVD, has been reported to be activated during nerve injury (Santos-Nogueira et al., 2015), and it transmits pro-inflammatory signals (Plastira et al., 2016). *DLG2*, which was downregulated in hypothalamus tissues with MD (Supplementary Table 2), has been reported to be associated with interferon production (Ali et al., 2018). These results suggest that the systematic effects left by CVD and MD can still be identified in several post-mortem human tissues on the transcriptomic level; among these tissues, top DEGs were reported to have a link with inflammation, indicating that inflammation may play a role.

### Inflammatory Events Enriched in Differentially Expressed Genes

**FIGURE 3:**
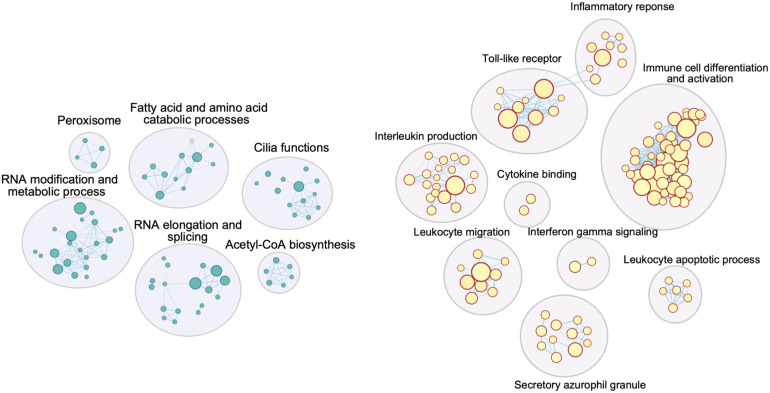
Cytoscape network image for GO terms significantly enriched in DEGs from the spinal cord in the CVD analysis. Similar GO terms (inner circles) were combined into groups (outer circles) by AutoAnnotate, summarizing labels generated from the app were further improved manually. Circles in blue (left) are GO terms enriched for downregulated DEGs, those in red (right) are GO terms enriched for upregulated DEGs (significant GO term’s cutoff: q-value < 0.1).

A functional overview can be gained through gene set enrichment analysis. CVD DEGs from the Adipose – Visceral, Artery – Tibial, Brain – Caudate, and Brain – Spinal cord; and MD DEGs from the Brain – Hypothalamus, Brain – Putamen, and Brain – Spinal cord were further analyzed using the Gene Set Enrichment Analysis method (Subramanian et al., 2005). A broad spectrum of Gene Ontology (GO) terms, with the top significantly enriched GO terms in the CVD spinal cord, is presented as an example in Figure 3.

Strikingly, upregulated genes in all four CVD tissues were significantly enriched in immunological events, for instance antigen binding, T cell proliferation, the interferon-gamma-mediated signaling pathway (Figure 3, Table 1), although the visceral adipose had only one significantly upregulated gene. This is consistent with a large body of evidence showing that inflammation plays a crucial role in cerebrovascular diseases. Inflammation can rupture the intracranial aneurysm wall (Tulamo et al., 2010), lead to secondary injury after an ischemic stroke (Ahmad and Graham, 2010), and impact the progression of symptomatic intracranial atherosclerosis (Arenillas et al., 2008). Inflammation has also been linked to blood–brain barrier dysfunction (de Vries et al., 2012) and tissue injury (Jin et al., 2010) in cerebrovascular diseases.

**TABLE 1:** Top three Gene Ontology (GO) terms with the highest normalized enrichment scores in tissues with many immunological terms.

GO term	NES	FDR q-val	FWER p-val
**Adipose – Visceral (CVD)**			
Extracellular matrix structural constituent conferring tensile strength	2.351	<0.001	<0.001
fc receptor mediated stimulatory signaling pathway	2.336	<0.001	<0.001
Antigen binding	2.258	0.005	0.012
**Artery – Tibial (CVD)**			
Immunoglobulin complex	3.722	<0.001	<0.001
Antigen binding	3.520	<0.001	<0.001
Co-translational protein targeting to membrane	3.386	<0.001	<0.001
**Brain – Caudate (CVD)**			
Positive regulation of T cell proliferation	2.980	<0.001	<0.001
Positive regulation of leukocyte cell-cell adhesion	2.934	<0.001	<0.001
T cell proliferation	2.929	<0.001	<0.001
**Brain – Spinal cord (CVD)**			
Response to interferon gamma	2.981	<0.001	<0.001
Leukocyte proliferation	2.936	<0.001	<0.001
Leukocyte cell cell adhesion	2.926	<0.001	<0.001
**Brain – Spinal cord (MD)**			
Antigen binding	−2.670	<0.001	<0.001
T cell receptor complex	−2.610	<0.001	<0.001
Immunoglobulin complex	−2.451	<0.001	<0.001

***NES**, normalized enrichment score; **FDR**, false discovery rate; **FWER**, familywise-error rate.*

For the MD tissues, inflammatory events only mainly enriched in downregulated genes only in the spinal cord (Table 1) and a few in upregulated genes in the hypothalamus (Supplementary Data Sheet 2). Likewise, depression has been associated with increased inflammatory activation in both the periphery and the central nervous system. Many antidepressant agents reduce inflammatory activation in immune cells and lower circulating inflammatory cytokine levels, supporting this association (Lee and Giuliani, 2019). Furthermore, it is worth mentioning that mitochondrial events and cellular respiration were significantly upregulated in the putamen. Since mitochondrial energy metabolism in the putamen has been reported to be highly correlated with emotional and intellectual impairment in Schizophrenics (Prince et al., 2000), it might also have some hidden links with depression as well. Another interesting result is that hypothalamus upregulated genes were mapped to terms related to cilia (Supplementary Data Sheet 1). There is still no obvious evidence connecting cilia with depression so far, but it is an underexplored area worth investigating (Pruski and Lang, 2019).

We further explored our differential expression results using Disease Ontology (Schriml et al., 2019) and Human Phenotype Ontology (Köhler et al., 2019) annotations. Top enriched diseases and human phenotypes are similar to the biological phenotypes found by our GO analysis (Supplementary Data Sheet 1). Immune responses and cerebrovascular lesions were significantly enriched in the upregulated genes of the CVD tissues. For example, six Disease Ontology terms—human immunodeficiency virus infectious diseases, temporal arteritis, alopecia areata, autoimmune thrombocytopenic purpura, intracranial aneurysms, and primary immunodeficiency diseases—were shared by all four CVD tissues’ overexpressed genes (Supplementary Figure 5), and they are all diseases associated with inflammation and cerebrovascular accidents.

Our enrichment results reinforce the strong evidence linking inflammation to CVD, as well as other interesting biological phenomena that probably have associations with CVD and MD in the analyzing tissues.

### Transcriptome-Wide Association Study

We then enquired whether the “transcriptomic scars” were associated with the subject’s genotype. TWAS is a powerful approach to prioritize target genes by combining genetic variants identified in GWAS with transcriptome data, and can help shed light on possible associations between genetic loci and human complex diseases. Here, we carried out TWAS with S-PrediXcan (Barbeira et al., 2018) on four public GWAS summary statistics datasets, in which human circulating levels of C-reactive protein (CRP) (Han et al., 2020), monocyte chemotactic protein-1 (MCP1), interleukin-6 (IL-6), and interferon-gamma (Ahola-Olli et al., 2017) were evaluated (Table 2). C-reactive protein is known as a systemic biomarker of inflammation and has been shown to be a CVD risk biomarker (Di Napoli et al., 2011) and to increase in patients with MD (Miller and Raison, 2016). Cytokines MCP1, IL-6 and interferon gamma have been reported to have a great probability of contributing to both CVD (Moreno et al., 2008; Seifert et al., 2014; Georgakis et al., 2019) and MD (Eyre et al., 2016; Franscina Pinto and Andrade, 2016; Hodes et al., 2016). Our aim was to find any overlaps between gene–tissue pairs identified from TWAS and from our DE analyses amongst the tissues with expression variance (including Adipose – Visceral, Artery – Tibial, Brain – Caudate, Brain – Hypothalamus, Brain – Putamen, and Brain – Spinal cord), plus whole blood, since circulating cytokine levels were measured in serum or plasma samples. There were 1,575 and 25 significant gene–tissue pairs passing the adjusted *P*-value threshold (*P*-value/number of genes) found in CRP and MCP1 data, respectively, regardless of tissue types (Supplementary Table 3). There were eight overlapping gene–tissue pairs (Table 3), amongst which *PPP1R18, RP11-238F2.1, FRK* had the same direction of variations but others had the opposite direction. All seven protein-coding genes were more or less related to immune responses. Specifically, it was demonstrated that complement gene *C2* was expressed in human post-mortem brain-derived cerebrovascular smooth muscle cells and may amplify the pro-inflammatory effects in brain vessels (Walker et al., 2008). The major histocompatibility complex class I chain-related gene A *MICA* is a highly polymorphic gene that encodes protein variants functioning in immune activation and surveillance; our results therefore indicate that there may be a link between *MICA* and depression.

**TABLE 2:** GWAS datasets used in this study.

Phenotype	Data source	Sample size
C-reactive protein (Han et al., 2020)	UK BioBank	418,642
MCP1 (Ahola-Olli et al., 2017)	The Cardiovascular Risk in Young Finns Study, FINRISK	8,293
IL-6 (Ahola-Olli et al., 2017)	The Cardiovascular Risk in Young Finns Study, FINRISK	8,293
Interferon gamma (Ahola-Olli et al., 2017)	The Cardiovascular Risk in Young Finns Study, FINRISK	8,293

**TABLE 3:** Overlaps of TWAS associations and DE genes.

Gene	Tissue	TWAS z-score	TWAS P.val	DE *t*-statistics	DE adj. P.val	Study
*C2*	Spinal cord	−6.43	1.27 × 10^–10^	5.36	7.93 × 10^–4^	CVD
*PSD4*	Spinal cord	−4.34	1.45 × 10^–5^	4.25	8.15 × 10^–3^	CVD
*PPP1R18*	Spinal cord	4.78	1.75 × 10^–6^	4.21	8.73 × 10^–3^	CVD
*PTPRJ*	Spinal cord	−5.02	5.10 × 10^–7^	3.82	0.017	CVD
*RP11-238F2.1*	Spinal cord	−5.07	4.08 × 10^–7^	−3.21	0.046	CVD
*FRK*	Spinal cord	−4.59	4.44 × 10^–6^	−3.15	0.050	CVD
*CD1E*	Whole blood	−8.74	2.25 × 10^–18^	4.43	0.040	CVD
*MICA*	Hypothalamus	−5.16	2.50 × 10^–7^	3.90	0.046	MD

## Discussion

Most CVD and MD transcriptome analyses (Grond-Ginsbach et al., 2008; Pantazatos et al., 2017; Kim et al., 2018; Cai et al., 2019; Zhu et al., 2019; Androvic et al., 2020; Wittenberg et al., 2020) are restricted to mouse models or a limited sample size of human expression data. In this study, we systematically analyzed expression data for over 16,000 non-diseased human samples across multiple tissues from GTEx, investigating global transcriptomic alterations on the human body in cases with a history of CVD or MD. We first built a linear mixed model and applied it to the expression data. Dozens to hundreds of differentially expressed genes were identified in the visceral adipose, tibial artery, caudate, and spinal cord for CVD, and in the hypothalamus, putamen, and spinal cord for MD. Furthermore, functional enrichment analysis showed that a large number of annotations pertaining to inflammatory responses were enriched in upregulated CVD DEGs from all four tissues, and that MD DEGs were mostly associated with neurological and metabolic events. Our results suggest that the long-term sequelae of cerebrovascular accidents and depressive symptoms can still be reflected in post-mortem samples, and that inflammation may be maintained for a period of time after CVD onset.

A growing body of evidence indicates that inflammation not only contributes to the initiation and development of CVD (Lakhan et al., 2009; Gu et al., 2019), it also persists globally in the brain for the long-term after CVD (Shi et al., 2019). Neuroinflammation followed by cerebrovascular accidents may promote recovery and further injury, playing both beneficial and detrimental roles (Jayaraj et al., 2019). A large-scale GWAS discovered one genetic variant (rs1842681) in the gene *LOC105372028* associated with post-stroke outcomes (Söderholm et al., 2019). Furthermore, proteomic studies of post-stroke depression (PSD) reveal that immune dysfunction in stroke survivors is associated with PSD (Zhan et al., 2014; Nguyen et al., 2016). The connection between inflammation and depression is undeniable (Dantzer et al., 2008; Miller and Raison, 2016). Unlike the CVD results, only a small portion of the MD DEGs were enriched in immune responses, while the top ones are mostly non-coding RNAs (Supplementary Table 2). This is reasonable since MD is highly heterogeneous (Goldberg, 2011) and not all individuals exposed to inflammatory challenges develop depression. Still, inflammatory responses that occur before and after cerebrovascular accidents or depression are very complicated, and the underlying mechanism is yet to be elucidated.

Our study also provides evidence of the general and potential long-term effects left by cerebrovascular events and depression from the transcriptomic aspect. A great number of DEGs were identified in some of the tissues, especially the brain, indicating that gene expression levels were alternated since CVD/MD onset. Non-brain tissues with significant CVD DEGs are related to vascular diseases and may pose risks to CVD. To be more specific, adipose tissue and its secreted inflammatory proteins contributed to obesity-associated vasculopathy and cardiovascular risk (Berg and Scherer, 2005), and they may contribute to CVD as well. Peripheral arterial disease occurring in the tibial artery shared similar risk factors with CVD (Banerjee et al., 2010). Moreover, DEGs such as *CHI3L1* and *LPAR5* may reveal possible mechanisms for post-CVD outcomes, but further experiments are necessary for validation. Interestingly, the hypothalamus had the highest number of MD DEGs, which is compatible with one of the most enduring and replicated findings in psychiatry — the activation of the hypothalamic-pituitary-adrenal axis in a subset of MD patients. The identified DEGs may play a role in the neuroendocrine function of the hypothalamus. The pathways enriched in putamen positive DEGs were mainly about mitochondrial functions and the electric transport chain, which replicates previous results (Sacchet et al., 2017) and provides new insights into the effects of depression. Apart from these, other tissues have a relatively small number of DEGs reported. The reasons could be (1) small sample sizes of tissues that limit the statistical power to detect any differences; (2) tissue specificity, in other words, the studied diseases (CVD and MD) may raise only a modest effect on the gene expression of those tissues. In addition, the effect may get compensated gradually or last not long enough to be detectable at the end of life. Further studies are needed to elucidate these points.

Only a few DEGs identified by our linear model overlapped with genes prioritized by TWAS for selected cytokine phenotypes. This was expected and is probably due to the small fraction of genetic risk factors shared by complex diseases and these circulating cytokine levels. Additionally, only about 11 percent of heritability was explained by bulk tissue expression quantitative trait loci, according to this study (Yao et al., 2020). Therefore, possible long-term transcriptomic alterations across tissues and organs are probably caused by external factors such as lifestyle and the social and physical environment. Nevertheless, we used bulk RNA-seq for our analyses, and further utilizing techniques with higher resolution, such as single-cell sequencing and cell-type decomposition from bulk sequencing, could reveal more precise signals on specific cell types.

## Conclusion

This study reveals molecular signatures of chronic effects and damage on multiple tissues potentially contributed by two types of complex diseases (CVD and MD) and associated factors. These signatures may be linked to inflammation and other disease-related pathways. Together, these results indicate that suffering from a complex disease can cause a tissue-wide impact on the transcriptomes, and they also suggest that treatment to attenuate inflammation may improve the body’s health in patients recovering from CVD. Our study not only provides insights into these disease mechanisms but also offers a possible route to studying the long-lasting changes caused by chronic diseases on multiple tissues or organs.

## Materials and Methods

### GTEx Data

Multi-tissue RNA-seq data were collected from the GTEx project (Aguet et al., 2020) v8 release (dbGaP: phs000424.v8.p2). The genes and samples were filtered and quantile-normalized in a tissue-aware manner, as described in the Yet Another RNA Normalization (YARN) pipeline paper (Paulson et al., 2017).

Subjects with an explicitly reported medical history of cerebrovascular disease (MHCVD, phv00169142.v8.p2) or major depression (MHDPRSSN, phv00169145.v8.p2) were considered in this study. We removed subjects with missing values in their Hardy scale (DTHHRDY), ischemic time (SMTSISCH), or batch ID (SMNABTCH). All cell lines and tissues with less than 12 samples with a history of CVD or less than 10 samples with a history of MD were excluded from our analyses. Finally, we used a total of 16,412 human post-mortem samples (1,498 with and 14,914 without a history of CVD), covering 46 tissues from 928 subjects (99 with and 829 without a history of CVD) in the CVD analysis (Supplementary Figure 1); and a total of 16,221 samples (1,602 with and 14,619 without a history of depression) across 45 tissues, including at least 10 samples with a history of MD, from 926 subjects (91 with and 835 without a history of MD) (Supplementary Figure 2).

### Differential Expression Analysis

Differential expression analysis between the samples with and without a history of CVD/MD was conducted using the voom-limma pipeline (Law et al., 2014; Ritchie et al., 2015). Briefly, RNA-seq read counts were transformed to log counts per million (log-cpm) with associated precision weights to stabilize the variance in the data using the *voom* function, followed by linear model fitting and the empirical Bayes procedure. According to the paper (Somekh et al., 2019), the multivariate linear regression model that adjusted for known confounders outperforms other methods correcting for hidden confounders, which may remove some of the desired biological signals. Hence, we adopted the linear regression model but replaced the experimental batch (*SMGEBTCH*) with another batch information (*SMNABTCH*). This model fits for gender (*GENDER*), the interval between the time of the donor’s death and the sample collection (*SMTSISCH*), age (*AGE*), the type of nucleic acid isolation batch (*SMNABTCH*), the type of death (*DTHHRDY*), as well as the variables of our interest—the medical history of CVD (*MHCVD*) and MD (*MHDPRSSN*)—for the gene expression data (*Y*):

*Y* ∼β*_1_GENDER* + β*_2_SMTSISCH* + β*_3_AGE* + β*_4_SMNABTCH* + *β_5_DTHHRDY* + *β_6_MHCVD* + *ε*

*Y* ∼*β_1_GENDER* + *β_2_SMTSISCH* + *β_3_AGE* + *β_4_SMNABTCH* + *β_5_DTHHRDY* + *β_6_MHDPRSSN* + *ε*

The *GENDER* term was removed from sex-specific tissues, and the *SMNABTCH* term was removed from tissues in only one batch. *P*-values from the regression model were adjusted for multiple testing using the Benjamini-Hochberg method (Benjamini and Hochberg, 1995).

### Functional Enrichment Analysis

Pre-ranked Gene Set Enrichment Analysis (GSEA) (Subramanian et al., 2005) was conducted with gene lists ranked by the *t*-statistics from the results of our DE analyses, with default program parameters and a default background set on GSEA v4.0.1. The Gene Matrix Transposed (GMT) files of Gene Ontology were obtained from the Molecular Signatures Database v7.1. Disease Ontology (Schriml et al., 2019) data were downloaded from the Alliance of Genome Resources^1^. Human Phenotype Ontology (Köhler et al., 2019) annotations were acquired from the website^2^. The Cytoscape (Shannon et al., 2003) figure (Figure 3) was generated with the AutoAnnotate application (Kucera et al., 2016), and auto-generated summarizing labels were further improved manually.

### Association Detection From GWAS Summary Statistics

GWAS summary statistics datasets were downloaded from the NHGRI-EBI GWAS Catalog (Buniello et al., 2019) for study GCST009777 (Han et al., 2020) and study GCST004421 (Ahola-Olli et al., 2017) on 19/10/2020. These GWAS datasets examined biomarkers of inflammatory responses, and they were obtained from Caucasian subjects (Table 2). Gene expression variation was inferred using S-PrediXcan (Barbeira et al., 2018) with GTEx v8 elastic-net prediction models^3^ for the four tissues with expression variation between CVD and non-CVD cohorts: Adipose – Visceral, Artery – Tibial, Brain – Caudate, and Brain – Spinal cord; and the three tissues with expression variation between MD and non-MD cohorts: Brain – Hypothalamus, Brain – Putamen, and Brain – Spinal cord. We ran S-PrediXcan on these tissues one by one in each phenotype. Tissue–gene pairs with *P*-value < 0.05/(number of tested genes) were considered as significant.

## Availability of Data and Materials

This project is under the approval of access request #84958 for the dataset General Research Use in Genotype-Tissue Expression (dbGaP: phs000424). The GTEx data were downloaded from dbGaP.

## Code Availability

GitHub repository: https://github.com/cyclab/GTEx-Complex-Diseases.

## Data Availability Statement

GTEx open-access data can be found on the GTEx Portal (https://gtexportal.org/home/datasets). GTEx protected data are available via dbGaP (accession phs000424.v8).

## Ethics Statement

This study involving reanalysis of the GTEx data was reviewed and approved by NCBI dbGaP (Project #22839).

## Author Contributions

C-LP and C-YC designed the study, wrote, and reviewed the manuscript. C-LP analyzed the data and drafted the manuscript. C-YC acquired the funding. Both contributed to the article and approved the submitted version.

## Conflict of Interest

The authors declare that the research was conducted in the absence of any commercial or financial relationships that could be construed as a potential conflict of interest.

## Footnotes

1 https://build.alliancegenome.org

2 https://hpo.jax.org/app/download/annotation

3 http://predictdb.org/

## Supplementary Material

Supplementary Figure 1 The number of samples with (MHCVD = 1) and without (MHCVD = 0) cerebrovascular disease history in each selected GTEx tissue.

Supplementary Figure 2 The number of samples with (MHDPRSSN = 1) and without (MHDPRSSN = 0) major depression history in each selected GTEx tissue.

Supplementary Figure 3 The distribution of age, BMI, sex, race in cohorts with (MHCVD = 1) and without (MHCVD = 0) cerebrovascular disease history.

Supplementary Figure 4 The distribution of age, BMI, sex, race in cohorts with (MHDPRSSN = 1) and without (MHDPRSSN = 0) major depression history.

Supplementary Figure 5 Venn diagram of **(A)** significant Disease Ontology (DO) terms enriched by upregulated DEGs, **(B)** significant DO terms enriched by downregulated DEGs, **(C)** significant Human Phenotype Ontology (HPO) terms enriched by upregulated DEGs, **(D)** significant HPO terms enriched by downregulated DEGs in four CVD tissues. The threshold is FDR.q.val < 0.05.

Supplementary Table 1 Significantly differentially expressed genes in CVD analysis.

Supplementary Table 2 Significantly differentially expressed genes in MD analysis. All limma-voom differential expression results, including limma reports, MA plots, and volcano plots, can be found at: https://doi.org/10.5281/zenodo.5075921.

Supplementary Table 3 Significant TWAS associations.

Supplementary Data Sheet 1 All GSEA results.

Supplementary Data Sheet 2 All TWAS results.
