# Supplementary material for: Exploring the Impact of Cerebrovascular Disease and Major Depression on Non-diseased Human Tissue Transcriptomes
Source: Front Genet. 2021 Jul 19;12:696836. doi: 10.3389/fgene.2021.696836 (PMC8327210; doi:10.3389/fgene.2021.696836)
Supplement: Supplementary Figure 1 — The number of samples with (MHCVD = 1) and without (MHCVD = 0) cerebrovascular disease history in each selected GTEx tissue. [file Presentation_1.pdf]

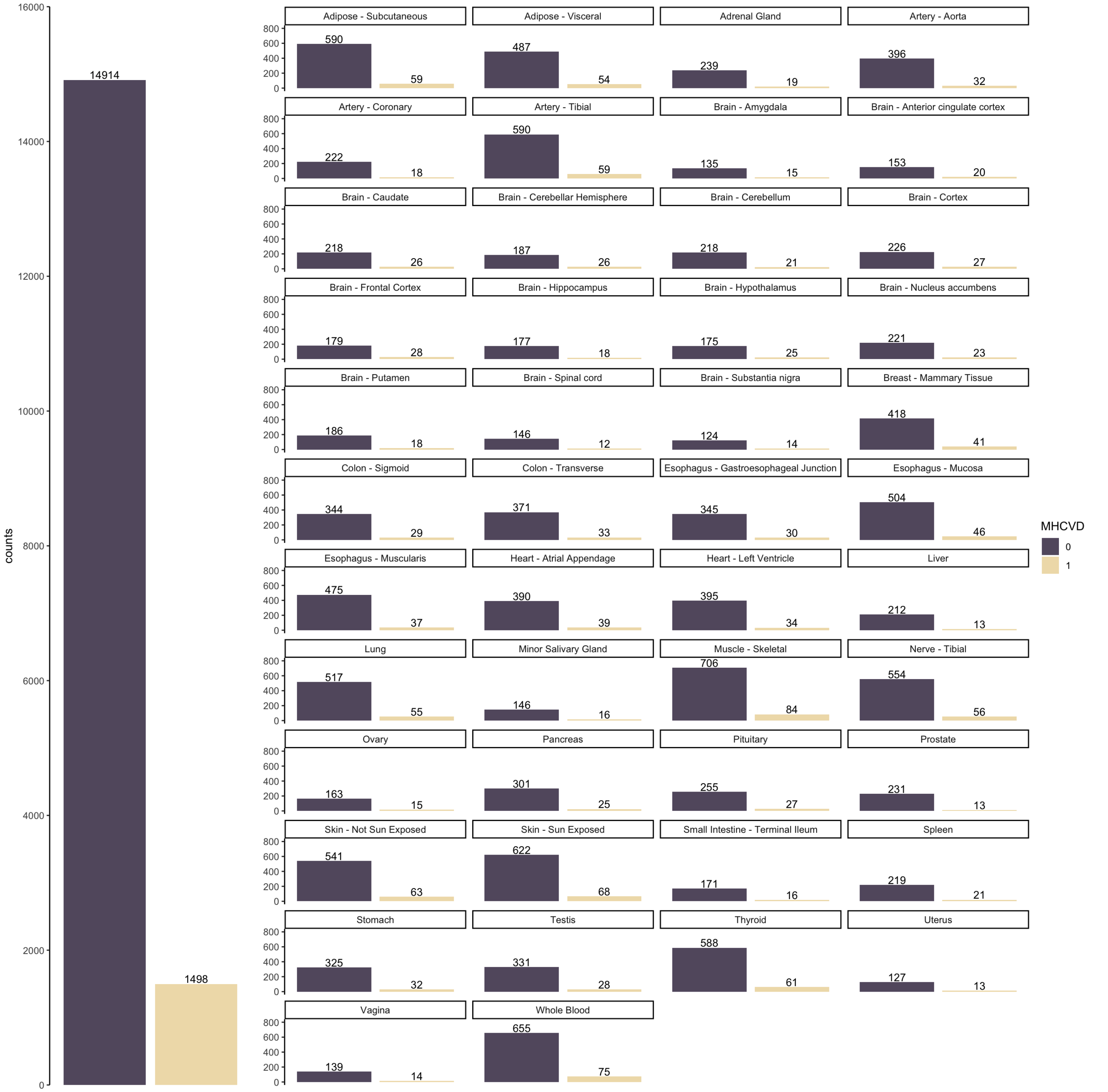

Supplementary Fig. S1 The number of samples with (MHCVD = 1) and without (MHCVD = 0) cerebrovascular disease history in each selected GTEx tissue.

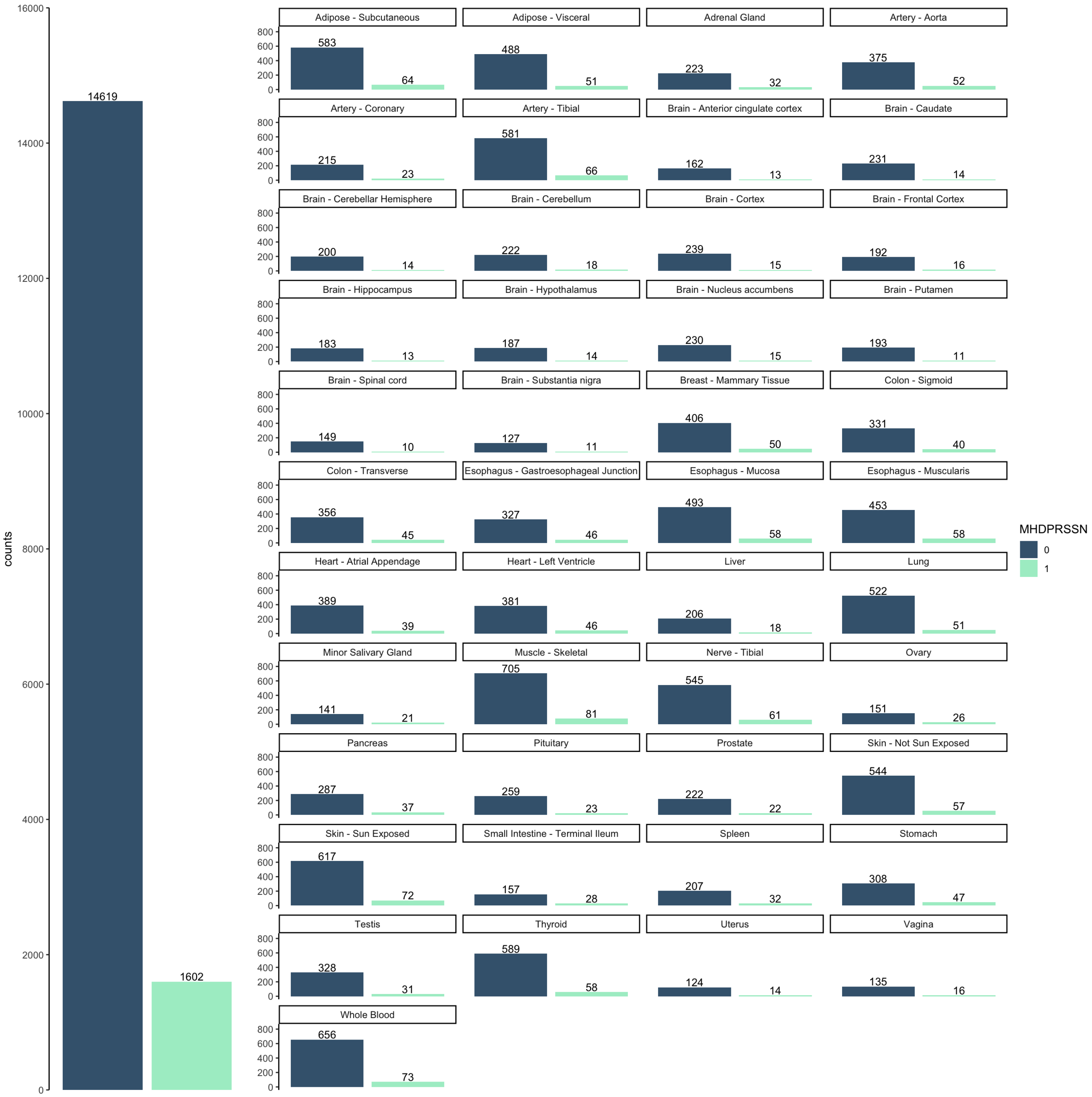

Supplementary Fig. S2 The number of samples with (MHDPRSSN = 1) and without (MHDPRSSN = 0) major depression history in each selected GTEx tissue.

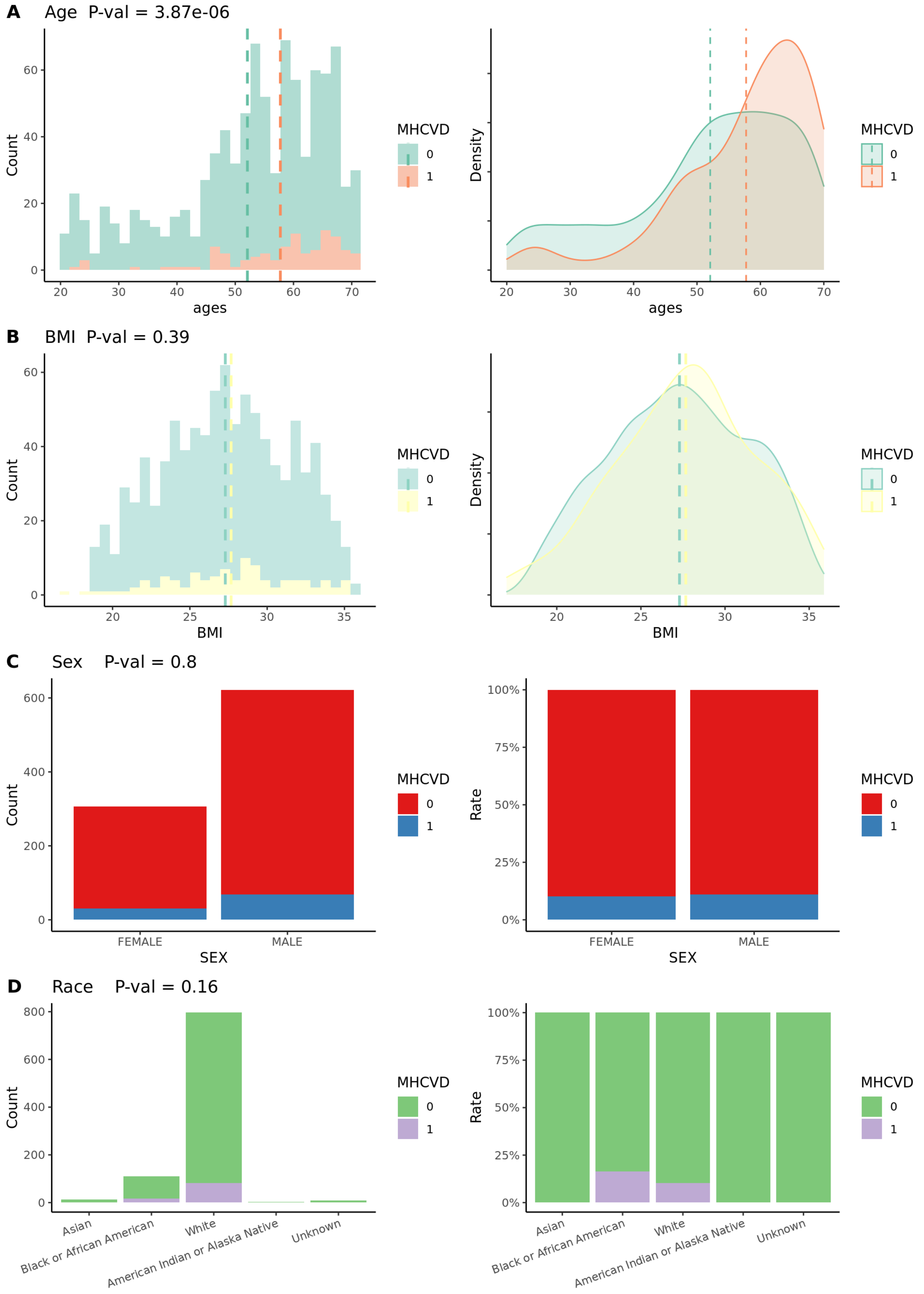

Supplementary Fig. S3 The distribution of age, BMI, sex, race in cohorts with (MHCVD = 1) and without (MHCVD = 0) cerebrovascular disease history.

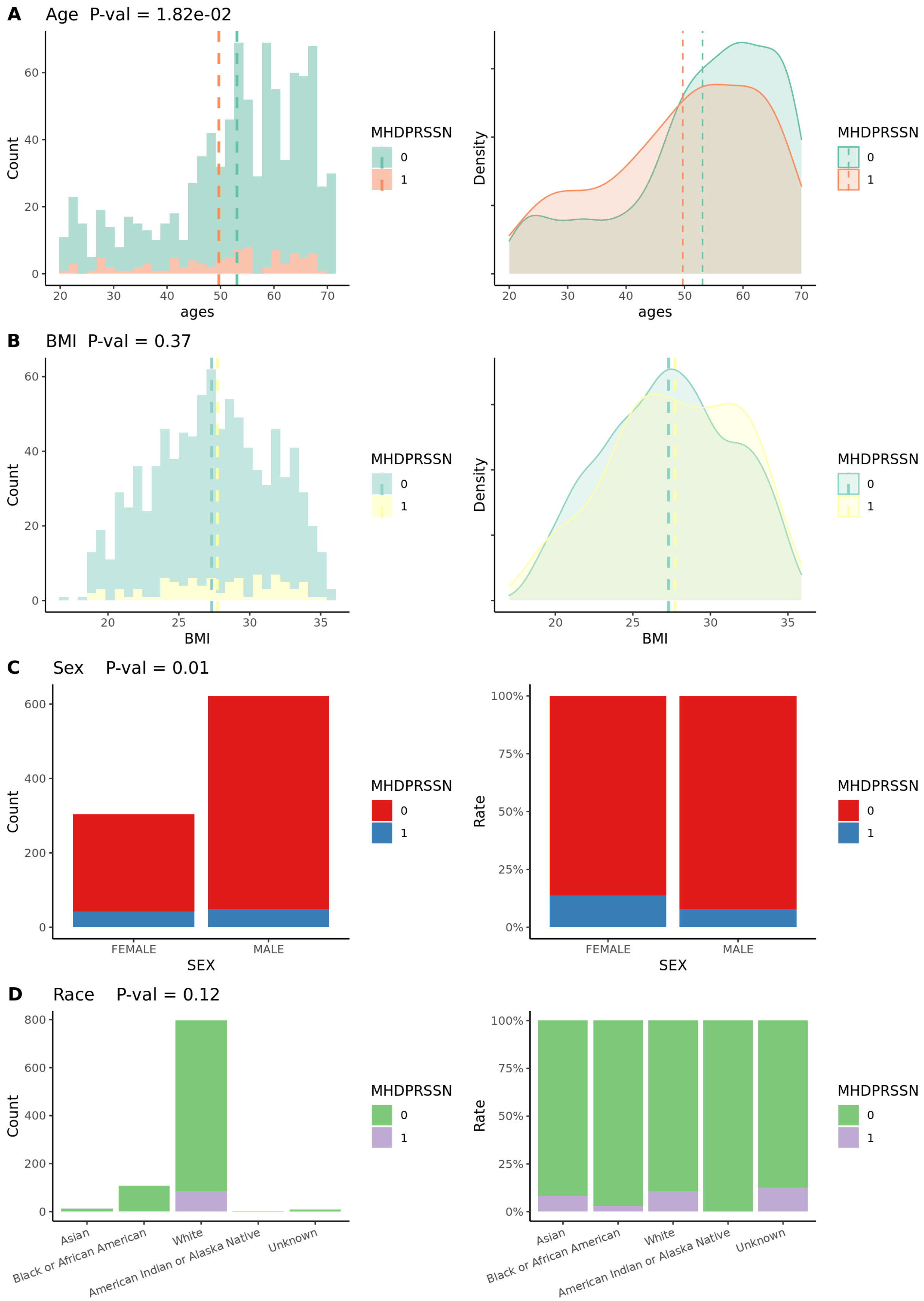

Supplementary Fig S4. The distribution of age, BMI, sex, race in cohorts with (MHDPRSSN = 1) and without (MHDPRSSN = 0) major depression history.

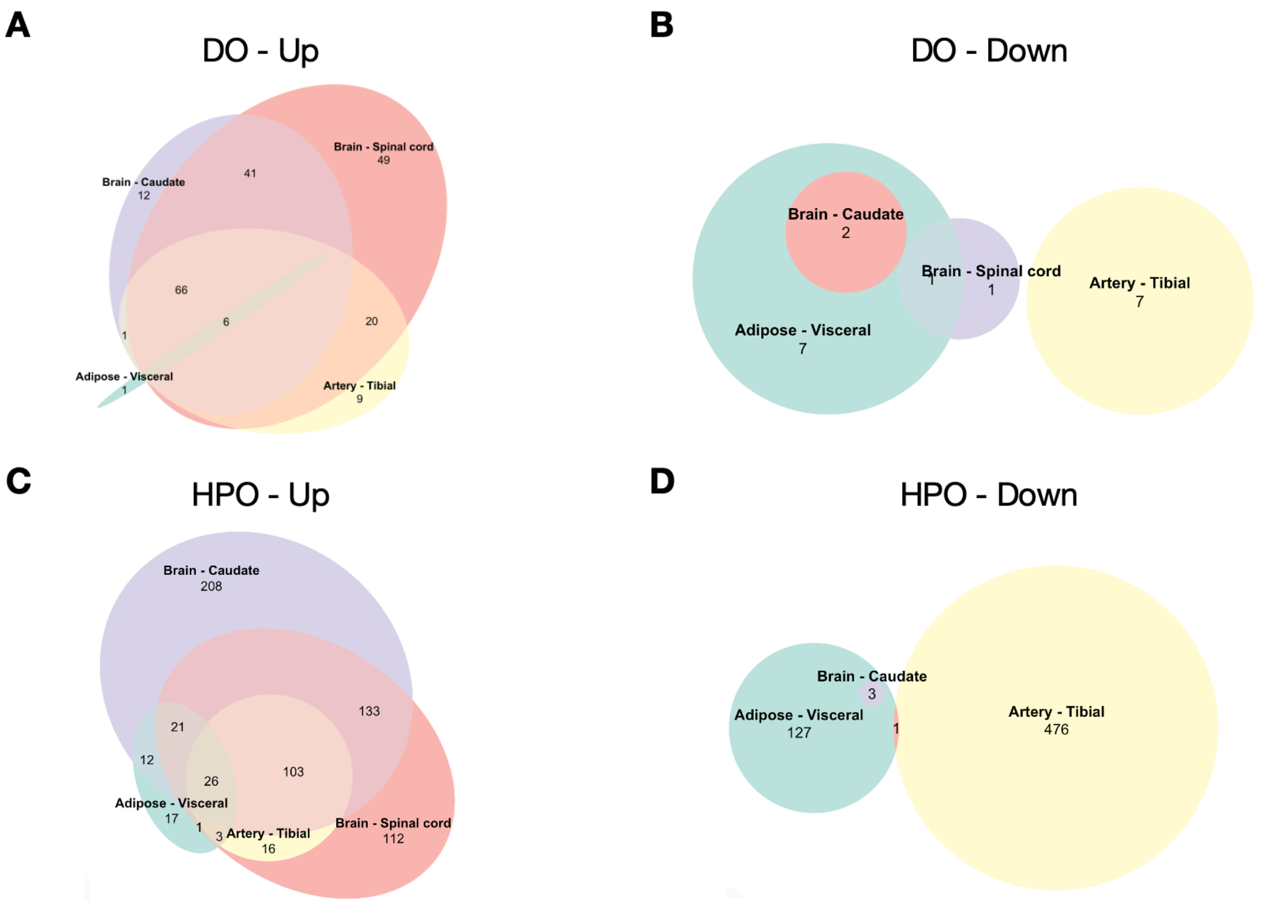

Supplementary Fig S5. Venn diagram of (A) significant Disease Ontology (DO) terms enriched by upregulated DEGs, (B) significant DO terms enriched by downregulated DEGs, (C) significant Human Phenotype Ontology (HPO) terms enriched by upregulated DEGs, (D) significant HPO terms enriched by downregulated DEGs in four CVD tissues. The threshold is FDR.q.val < 0.05.
